# The Complex Roles of Macrophages in Sjögren’s Syndrome: A Narrative Review

**DOI:** 10.7759/cureus.89486

**Published:** 2025-08-06

**Authors:** Yuan Ren, Ge-Dan Cui, Cong Wu, Tao Hu, Xiao-Yan Li, Jian-Mei Hao

**Affiliations:** 1 Department of Hepatology, Xi'an Hospital of Traditional Chinese Medicine, Xi'an, CHN; 2 Department of Pulmonology, Xi'an Hospital of Traditional Chinese Medicine, Xi'an, CHN; 3 Department of Hepatology, Xi'an Hospital of Traditional Chinese Medicine, xi'an, CHN

**Keywords:** lacrimal glands, lymphocytes, macrophages, salivary glands, sjögren's syndrome

## Abstract

Sjögren's syndrome (SS) is an autoimmune disease characterized by the destruction of the structure and function of exocrine glands (EGs) such as lacrimal glands (LGs) and salivary glands (SGs). During the pathogenesis, various immune cells such as lymphocytes, dendritic cells, and macrophages are activated, which together maintain the pro-inflammatory environment of the EGs. As an important immune cell linking innate and specific immunity, macrophages have both functions of phagocytosis and antigen presentation. Studies in animal models and SS patients have suggested that macrophages play an important role in the clearance of apoptotic cells and the activation of lymphocytes in the EGs of SS patients. This narrative review highlights the roles of macrophages in the development of SS, with the aim of providing a comprehensive reference for future research.

## Introduction and background

Sjögren's syndrome (SS) is the third most common autoimmune disease, following systemic lupus erythematosus (SLE) and rheumatoid arthritis (RA) [1]. It primarily manifests in two forms: primary and secondary. Secondary SS is defined as a condition where patients develop characteristics like oral and ocular dryness on the basis of a confirmed diagnosis of other autoimmune diseases such as systemic lupus erythematosus, rheumatoid arthritis and systemic sclerosis. In contrast, primary Sjögren's syndrome (PSS) patients do not have other autoimmune diseases that could potentially trigger SS [2]. Currently, the global prevalence of SS is estimated to range from 0.01% to 0.72%. The condition is more prevalent in women, with a male-to-female ratio of approximately 1:9 [3,4]. Most female patients develop the disease during menopause, with an average age of onset around 55 years [5]. The main pathological change of SS is the massive infiltration of lymphocytes into exocrine glands (EGs) such as thelacrimal glands (LGs) and salivary glands (SGs), which leads to the destruction of the structure and function of EGs, thereby resulting in sicca symptoms such as xerostomia and keratoconjunctivitis sicca [6]. As the disease advances, manifestations may also emerge in other organs such as the kidneys and lungs [7,8]. Therefore, in clinical practice, minor SGs biopsy is usually performed on patients with suspected SS. A focus score ≥ 1 or a Chisholm-Mason score > 2 is considered an important basis for diagnosis [9].

Previous studies hold that the infiltration of CD4+ T lymphocytes and B lymphocytes into EGs, where they form ectopic germinal centers (EGC). EGC are functional structures formed by the abnormal aggregation of immune cells in the EGs of SS patients, and serve as a key hub for the production of autoantibodies such as anti-SSA/Ro and anti-SSB/La and the maintenance of chronic inflammation. The excessive activation of B lymphocytes has been identified as a hallmark feature of SS [10-12]. Hence, so far, researchers have paid more attention to lymphocytes while less attention to macrophages in the study of SS. In fact, macrophage infiltration even precedes lymphocytic infiltration in SS patients' EGs [13,14]. Macrophages, a professional antigen-presenting cell, mainly establish the link between innate and adaptive immunity. Innate immunity is a natural defense mechanism formed by organisms during long-term evolution, while adaptive immunity is a targeted defense mechanism gradually developed by the body after exposure to specific pathogens or antigens. Macrophages participate in inflammatory responses by secreting inflammatory cytokines and regulating the activity of other immune cells [15]. A study by Kramer et al. showed that in the advanced disease, the percentage of macrophages in the SGs of non-obese diabetic (NOD) mice was three times that of the control group [16]. Furthermore, another important study has shown that in the minor SGs of PSS patients, the expression of M1 macrophages was increased, while the expression of M2 macrophages was decreased [17]. Similarly, the expression of inducible nitric oxide synthase (iNOS), a marker of M1 macrophages, was significantly upregulated; however, the expression of CD206, a marker of M2 macrophages, was downregulated in the LGs of rabbits with autoimmune dacryoadenitis [18]. Interestingly, reducing the number of macrophages has been shown to improve the structure and function of LGs in SS mice [19]. The above studies indicate that macrophages are important immune cells that mediate inflammatory responses in the EGs in SS. Therefore, studies targeting macrophages may provide a new direction for revealing the pathogenesis and the treatment of SS. However, there is a paucity of narrative reviews regarding the roles of SS-related macrophages. To provide a detailed reference for future research, we completed this narrative review.

Methodology

The main purpose of this narrative review is to comprehensively summarize the published articles on the mechanism of action of macrophages in the pathogenesis of SS from 1995 to 2025. We searched multiple databases such as PubMed, Web of Science, and Google Scholar using "macrophages" and "Sjögren's syndrome" as keywords. The inclusion criteria were open-access scientific publications, human, animal, and cellular studies related to macrophages and SS, as well as reviews relevant to the topic, with the language being English. The exclusion criteria were studies with flawed design, insufficient data, or outdated information. Through the above methods, we initially obtained 274 articles, and after subsequent exclusion, we finally included a total of 96 manuscripts that are most relevant to the theme of this article.

## Review

Macrophage infiltration of EGs occurs in the early stage of SS

Studies have shown that CD68 + macrophage infiltration in the SGs of NOD mice occurs as early as eight weeks old, which is earlier than lymphocyte infiltration (at 12 weeks old) and the decline in salivary function (at 16 weeks old) [13]. More importantly, the number of infiltrating macrophages has gradually increased over time and is thought to be positively correlated with disease severity [16]. Studies have shown that the massive infiltration of macrophages in the EGs of SS patients may provide the necessary conditions for subsequent lymphocyte activation. Firstly, as antigen-presenting cells, macrophages can activate CD4+ lymphocytes by expressing major histocompatibility complex II (MHC-II) [20]. Secondly, macrophages highly express C-C motif chemokine ligand (CCL)5, which directly recruits T cells to the EGs by binding to the CCR5 receptor on the surface of T cells, thereby exacerbating glandular damage [21]. 

**Figure 1:**
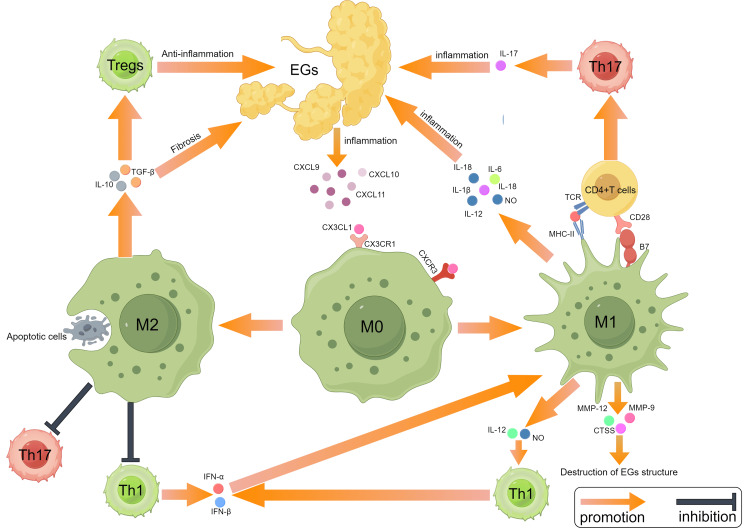
The contributions of macrophages in Sjögren's syndrome EGs: exocrine glands Image created by the authors.

At present, several hypotheses have been put forward to account for the gradual increase in the number of macrophages in the EGs of SS patients. To begin with, elevated apoptotic cells were observed in the EGs in eight- to 10-week-old NOD mice, a time when lymphocyte infiltration had not yet occurred [22]. Therefore, macrophages are recruited to the exocrine glands to clear apoptotic cells. Second, another important study also demonstrated that the high expression of CX3CL1 could be observed in the SGs of 10-week-old NOD mice, which is at the pre-SS disease or subclinical stage [23]. CX3CL1, the sole member of the C-X3-C chemokine family, exists in two forms: membrane-bound and soluble. Significantly, both forms of CX3CL1 can bind to the C-X3-C motif chemokine receptor 1 (CX3CR1), which is highly expressed on monocytes [11]. Thus, highly expressed CX3CL1 allows a large number of peripheral CX3CR1 + monocytes to migrate to EGs and differentiate into macrophages [24]. The contributions of macrophages to SS are illustrated in Figure 1 and elaborated upon in the subsequent discussion.

Macrophage phenotypes in SS

Macrophages are mainly differentiated from monocytes in response to the stimulation of macrophage colony-stimulating factor (M-CSF) or granulocyte-macrophage colony-stimulating factor (GM-CSF) [25]. Macrophages are divided into classically activated macrophages (M1) and alternatively activated macrophages (M2). M1 macrophages highly express cytokines such as interleukin (IL)-1β, IL-6, and tumor necrosis factor alpha (TNF-α), and exacerbate glandular destruction through pro-inflammatory responses. In contrast, M2 macrophages highly express IL-10 and transforming growth factor beta (TGF-β), exerting anti-inflammatory and glandular repair functions [17]. Two types of macrophages coexist in the EGs of SS patients, jointly maintaining the immune microenvironment of the EGs.

M1 macrophages and their expressed cytokines

The polarization of M1 macrophages is driven by stimulation with extracellular pathogen-associated molecular patterns (PAMPs) or interferon gamma (IFN-γ). Once activated, macrophages immediately express high levels of pro-inflammatory cytokines such as IL-1β, IL-6, IL-18, TNF-α, nitric oxide (NO), and reactive oxygen species (ROS) to participate in autoimmune diseases.

The expression of IL-1β is upregulated in SS-associated macrophages [26]. A study has shown that the increased expression of IL-1β in the SGs of NOD mice precedes lymphoid aggregation and secretory dysfunction of acinar cells [27]. This seems to imply that IL-1β expressed by macrophages is a key factor mediating the early inflammatory response and lymphocyte infiltration in the SGs. Current studies indicate that the role of IL-1β in SS may be concentrated in the following aspects. First, IL-1β can promote the differentiation of Th17 cells, and the increase in Th17 cells in SS patients is positively correlated with the severity of the disease [28]. Second, as two proinflammatory cytokines expressed by M1 macrophages, IL-1β and TNF-α exhibit synergistic effects in multiple functions. For instance, IL-1β and TNF-α can promote the overexpression and abnormal accumulation of mucin in the SGs of SS, thereby leading to SGs dysfunction [29]. Interestingly, apart from exerting a synergistic effect with IL-1β, TNF-α is also considered in some studies to be capable of affecting salivary secretion independently, which is mainly related to the downregulation of the expression of tight junction protein 1 in the SGs [30]. Although IL-1β and TNF-α are of great significance in the pathogenesis of SS, clinical trials using IL-1β or TNF-α blockers for the treatment of SS have not shown efficacy. This may indicate that we need to further explore their specific mechanisms and therapeutic approaches in SS in the future. 

IL-6 levels are significantly increased in the tear fluid of SS patients and are considered to be positively correlated with disease severity [31]. IL-6 is essential for Th17 cells differentiation, and it also inhibits regulatory T cells (Tregs) differentiation [32]. Blocking IL-6 signaling has been shown to mitigate SGs injury in NOD mice by correcting the imbalance between Tregs and Th17 cells [33]. Of course, IL-6 not only participates in the regulation of SS-related immune cells but also promotes TGF-β-mediated SGs fibrosis by enhancing the expression of vimentin and collagen type I [34].

NO is a gaseous signaling molecule expressed by M1 macrophages, whose synthesis involves iNOS. Studies have shown that the expression levels of iNOS and NO in lacrimal acinar cells of SS are much lower than those in infiltrating macrophages [35]. Long-term exposure to NO significantly increases the risk of developing SS [36]. The specific reasons mainly include the following: First, NO inhibits cell growth and division by reducing the activity of DNA synthase and mitochondria. Second, NO relaxes pericytes and myoepithelial cells. Decreased contractile function of myoepithelial cells leads to secretory dysfunction of EGs [37]. Third, overproduction of NO leads to massive death of lacrimal cells [35].

The strongly increased expression of IL-18 in the EGs of SS patients and mice has been confirmed in numerous studies [38,39]. A study suggests that IL-18 expressed by macrophages may be involved in the formation of EGC in the SGs [40]. Autoantibodies such as anti-SSA/Ro and anti-SSB/La have been shown to be mainly produced in EGs with EGC [41]. In fact, these antibody-positive patients had higher levels of IL-18 compared to antibody-negative patients [42]. Therefore, the highly expressed IL-18 by macrophages may be closely related to the formation of EGC and the production of anti-SSA/Ro and anti-SSB/La. Targeting the IL-18 signaling axis may inhibit the abnormal immune response in the SGs, and studies have explored the application of such strategies in the treatment of SS [39].

IL-12 is another pro-inflammatory factor expressed by macrophages. Several studies have shown that IL-12 is highly expressed in the peripheral blood and SGs of SS patients [43,44]. This indicates that IL-12 may have an important impact on the pathogenesis of SS. At present, one study has shown that IL-12 can exacerbate SS by inducing myeloid-derived suppressor cells, but this is far from sufficient to elaborate on the specific role of IL-12 in SS, and more mechanisms need to be further explored in the future [45]. 

M2 macrophages and their expressed cytokines

The polarization of M2 macrophages is driven by IL-4 and IL-13, and is characterized by the marked expression of TGF-β and IL-10 [46]. The SGs of SS patients exhibit high expression of chemokines including CXCL9, CXCL10, and CXCL11 [47]. These elevated chemokines primarily mediate the migration of CXCR3+ T lymphocytes into the SGs [48]. Therefore, in some studies, treatment with anti-CXCR3 antibody has been shown to significantly reduce the infiltration of T lymphocytes in the SGs [49]. A recent study showed that the main immune cells expressing the receptor CXCR3 in the SGs of SS patients were CD80+ macrophages and CD163+ macrophages, rather than lymphocytes. CXCR3+ CD163+ macrophages, identified as M2 macrophages, exhibit notable anti-inflammatory properties. M2 macrophages maintain the homeostasis of EGs mainly by phagocytosing apoptotic cells and expressing anti-inflammatory cytokines. Interestingly, the number of M2 macrophages in the SGs and peripheral blood gradually decreased with disease progression [17]. Decreased M2 macrophages may be one of the reasons for inflammatory responses in EGs.

IL-10 is a cytokine with immunosuppressive functions and plays an important role in autoimmune diseases. Upregulating the expression of IL-10 can improve salivary flow rate and reduce inflammatory responses in the SGs [50]. The protective effect of IL-10 on the SGs is mainly reflected in two aspects. On the one hand, it can inhibit the differentiation of Th1 and Th17 cells by promoting STAT5 phosphorylation, thereby controlling the inflammatory response [51]. On the other hand, IL-10 can inhibit the apoptosis of SGEC by enhancing the expression of X-linked inhibitor of apoptosis protein (XIAP) [52].

Among the TGF-β isoforms (TGF-β1, -β2, and -β3), TGF-β1 plays a crucial role in the pathogenesis of SS. A study has shown that the expression level of serum TGF-β1 in SS patients increases significantly with the aggravation of disease severity [53]. This may be related to the pro-fibrotic effect of TGF-β1. Moreover, as one of the most important cytokines for M2 macrophages, TGF-β1 has a significant anti-inflammatory effect. TGF-β1 deficiency leads to marked immune cell infiltration, structural destruction of EGs, and consequent secretory dysfunction. A study has shown that female mice with knockout of the TGF-β1 receptor exhibit an increase in Th1 cells and related proinflammatory cytokines in the SGs [54]. Therefore, the anti-inflammatory effect of TGF-β1 seems to be related to the differentiation of T lymphocytes.

Other functional proteins expressed by macrophages

Calprotectin is a calcium-containing protein produced by macrophages. Studies have demonstrated that the expression of calprotectin is significantly elevated in SS patients, with its primary expression localized to the inflamed SGs [55]. Another study has shown that the highly expressed calprotectin in SS patients may mainly originate from macrophages that infiltrate the SGs [56]. More importantly, calprotectin has been shown to strongly induce immune cells to express IL-1β, IL-6, TNF-α, and IFN-γ, thereby participating in the inflammatory response of SGs in SS patients [57]. Therefore, some studies suggest that calprotectin has the potential to be a biological marker for SS [58]. However, regrettably, there are currently few studies on the relationship between calprotectin and the pathogenesis of SS, and more potential mechanisms need to be further explored in the future.

Vasoactive intestinal peptide receptors (VPAC) 1 and VPAC2 are expressed on SS-associated macrophages, with higher expression observed in M1 compared to M2 macrophages [59]. A study has shown that vasoactive intestinal peptide (VIP), an endogenous ligand for VPAC1 and VPAC2, can alleviate SS-related symptoms by inhibiting the PI3K/AKT pathway to restore the balance between Treg and Th17 cells [60]. Therefore, VIP has been reported as a candidate drug with anti-inflammatory and immunomodulatory properties for the treatment of SS. Interestingly, VPAC2 expression in SS-related macrophages was up-regulated, but macrophages still showed impaired clearance of apoptotic cells [61]. The main reason for this phenomenon may be the gradual decline of VIP expression with the SS development [62]. Therefore, VIP/VPAC plays an important role in the function of SS-related macrophages.

Function of macrophages in SS

Clearing Apoptotic Cells in EGs

Apoptotic cells, whether physiological or pathological, are eliminated by macrophages, thereby providing a suitable and essential microenvironment for synthesis and secretion of tears or saliva. M2 macrophage polarization is enhanced upon encounter with apoptotic acinar cells. The process by which M2 macrophages remove apoptotic cells is defined as efferocytosis, which is characterized by increased expression of anti-inflammatory factors and accelerated degradation of pro-inflammatory factors [63]. TAM receptors, a subfamily of receptor tyrosine kinases including Tyro3, Axl, and Mer, are expressed on the surface of macrophages and serve as key proteins in mediating efferocytosis [64]. Mer is divided into membrane-bound forms and soluble forms. Mer signaling pathway exerts a protective role in various autoimmune diseases. A study has shown that Mer knockout mice exhibit typical characteristics of SS, such as lymphocytic infiltration in the SGs and decreased salivation [65]. This may be because membrane-bound Mer is the core protein for macrophages to perform phagocytosis [66]. Finally, the phagocytosed apoptotic cells will be transported to the lysosomes in macrophages for digestion to ensure the homeostasis of the in vivo microenvironment and avoid inflammatory responses.

Maintaining the Inflammatory Microenvironment

Efferocytosis is one of the important means for macrophages to maintain homeostasis in EGs. Macrophage-mediated efferocytosis is regulated by Tregs. Unfortunately, the number of Tregs cells gradually decreases with the aggravation of SS [67]. In addition, metalloproteinase 17 (ADAM17), which is highly expressed in SS mice, is an important protein responsible for defective efferocytosis [65]. The coexistence of the above factors prevents apoptotic cells from being cleared by macrophages in time and develops secondary necrosis that is characterized by the leakage of double-stranded DNA (dsDNA) outside the cells. Undegraded dsDNA accumulates extensively in the EGs of SS patients, functioning as a signal to activate inflammasomes-intracellular multi-protein complexes that play an extremely important role in the pro-inflammatory effects of M1 macrophages. NOD-like receptor pyrin domain-containing 3 (NLRP3) is the primary inflammasome that undergoes hyperactivation by undegraded dsDNA in SS-associated macrophages [68]. NLRP3 and its related inflammatory factors, including caspase-1, IL-1β, and IL-18, have also been shown to be significantly upregulated in the EGs of SS patients [69]. Absent in melanoma 2 (AIM2), an IFN-γ-induced protein, is thought to play a key role in the inflammasome formation of macrophages induced by dsDNA. The expression of AIM2 is up-regulated by highly expressed dsDNA in SS patients [68,70,71]. The HIN200 domain of AIM2 binds to the recognized dsDNA, while the PYD domain of AIM2 binds to the PYD domain of the apoptosis-related speck-like protein ASC to form a caspase-1-activating inflammasome. On the one hand, activated Caspase-1 induces pyroptosis by activating GasderminD. On the other hand, activated Caspase-1 cleaves the precursors of IL-1β and IL-18 into active IL-1β and IL-18, which are released extracellularly to cause an inflammatory response [72]. Therefore, blocking the AIM2 signal may significantly reduce the activation of inflammasomes in macrophages.

Promoting Lymphocyte Maturation

T cell activation is tightly regulated by costimulatory molecules. When MHC-II of APCs is recognized by T cell receptors (TCR), the costimulatory molecule CD28 expressed on the surface of T lymphocytes must bind to the costimulatory molecule B7 (CD80/CD86) expressed on the surface of APCs to provide the necessary signal stimulation for T lymphocytes activation and cytokine production [73]. More importantly, macrophages are key APCs that express the costimulatory molecules CD80/CD86. A study has shown that inhibiting the expression of CD80/CD86 on the surface of macrophages can significantly reduce lymphocyte infiltration in EGs, downregulate the expression of inflammatory factors such as TNF-α, IL-1β, and IL-6, thereby improving the function of SGs [74].

In addition to providing the necessary costimulatory molecules and antigen signals for the activation of lymphocytes, SS-associated macrophages are also the main cells expressing B-cell activating factor (BAFF) in the SGs [75]. BAFF has been proven in previous studies to be a key factor in promoting the maturation of B lymphocytes, inducing the synthesis of anti-Ro/SSA and anti-La/SSB [76-78]. In addition, BAFF can also strengthen the chemotactic effect of CXCL13 on B lymphocytes. This may be related to the simultaneous expression of CXCR5 and BAFFR, a BAFF-specific receptor, on B lymphocytes in the SGs of SS mice and humans [16]. A study indicates that intervention with Sandy-2, a BAFF-blocking monoclonal antibody, in NOD mice can significantly reduce the number of B lymphocytes in the SGs and improve their salivary flow [79]. Therefore, the expression of BAFF by macrophages is of crucial significance to the pathogenesis of SS, and BAFF-targeted therapy may become a new direction for the treatment of SS.

Promoting Lymphocyte Migration to EGs

The accumulation of macrophages in EGs precedes lymphocyte infiltration [13]. Those macrophages specifically mediate lymphocyte migration by expressing different chemokines. The study performed by Ushio showed that macrophages with highly expressed CD11b more strongly expressed CCL22 compared to macrophages with lowly expressed CD11b. C-C chemokine receptor 4 (CCR4), a receptor that recognizes CCL22, is primarily expressed on the surface of CD4+ T lymphocytes. Consequently, macrophages with high CD11b expression enhance the migratory activity of CD4+ T lymphocytes toward the SGs by activating CCR4 [80].

In addition, macrophages infiltrating the SGs also participate in the migration of B lymphocytes. Studies have shown that 74% of SS patients exhibited elevated CXCL13 in either serum or saliva, and elevated CXCL13 was demonstrated to be mainly expressed by macrophages infiltrating the SGs [16,40]. Its receptor, CXCR5, is mainly expressed on B lymphocytes and is absent in healthy salivary tissue. More importantly, both in vitro and in vivo experiments have confirmed this pathological mechanism: CXCL13, which is highly expressed in macrophages under the induction of IFN-α, leads to the accumulation of CXCR5+ B lymphocytes in the SGs [81]. Therefore, CXCL13 expressed by macrophages is a key cytokine responsible for the migration of B lymphocytes to the SGs and the formation of EGC [82].

Participating in EGs Fibrosis

The pro-fibrotic effect of macrophages in SS patients is primarily attributed to their high expression of TGF-β1. Notably, TGF-β1 has been implicated in sialadenitis, post-radiotherapy SGs dysfunction, and the pro-fibrotic pathogenesis of SS [83]. The serum level of TGF-β1 is significantly higher than that in the healthy control group, and it is positively correlated with disease activity and labial gland pathological grading in SS patients [53]. Increased expression of TGF-β1 promotes the differentiation of fibroblasts into myofibroblasts, which in turn leads to extracellular matrix (ECM) deposition and glandular fibrosis in the SGs of SS patients. Glandular fibrosis is an irreversible morphological change in the advanced stage of the disease, directly resulting in glandular atrophy and loss of function [84,85]. Studies have confirmed that the pro-fibrotic effect of TGF-β1 is related to its regulation of the SMAD/Snail signaling pathway, while inhibition of the TGF-β1 signal can effectively reduce the fibrosis of EGs in SS patients [86,87]. 

In addition, macrophages can also express matrix metalloproteinases (MMPs) and cathepsins. A study indicated that CD68+ macrophages highly express MMP-9 and MMP-12 in severely diseased SGs [88]. In addition to expressing MMP-9, macrophages in the LGs of mice also highly express cathepsin S and cathepsin H. Interestingly, cathepsin S is thought to be expressed exclusively by CD68 + macrophages, whereas cathepsin H can also be expressed by cells other than CD68 + macrophages, such as CD4 + T lymphocytes [89]. Although MMP-9, MMP-12, cathepsin S, and cathepsin H have been proven in previous studies to promote ECM degradation and thus possess certain anti-fibrotic effects, it is interesting that all the aforementioned studies indicate that the high expression of these proteins exacerbates the inflammatory response, structural damage, and fibrosis in the EGs. Secondly, CD68 is expressed in both M1 and M2 macrophages, and there is currently no research to determine whether MMP-9, MMP-12, cathepsin S, and cathepsin H are expressed in M1 or M2 macrophages. Therefore, the pro-fibrotic mechanism of macrophages in the EGs of SS patients needs further exploration. 

Macrophage-targeted therapeutic strategies

A growing body of research has found that macrophages may become a breakthrough in the treatment of SS. Currently, macrophage-based therapeutic strategies mainly include immunomodulatory therapy, novel cell therapy, molecular targeted therapy, and dietary therapy.

Immunomodulatory Therapy 

Mesenchymal stem cells (MSCs), multifunctional stem cells, have been shown to have the potential to treat Sjögren's syndrome [90]. The therapeutic effect of MSCs in autoimmune diseases is related to the induction of immune tolerance by affecting the polarization of macrophages and the expression of inflammatory cytokines [91]. The study conducted by Lu indicated that MSCs can significantly inhibit M1 macrophage polarization and promote M2 macrophage polarization by activating the AKT signaling pathway to treat rabbit autoimmune LGs inflammation [18]. Furthermore, another study has proven that dexamethasone can convert macrophages from the pro-inflammatory (M1) phenotype to the anti-inflammatory (M2) phenotype, thereby reducing acinar cell atrophy and fibrosis, but high-dose or long-term use may induce acino-ductal metaplasia [92]. Therefore, it is not suitable as a routine treatment for SS.

Novel Cell Therapy

Novel cell therapy refers to an innovative therapeutic method that, on the basis of traditional cell therapy, combines cutting-edge technologies such as gene editing, synthetic biology, and precise targeting to modify cells, so as to enhance therapeutic effects and reduce side effects. Its core purpose is to use "living cells" as therapeutic carriers to repair damaged tissues, regulate immune functions, or directly eliminate diseased cells [93]. Cytotoxic T lymphocyte antigen 4 (CTLA-4), as a key negative costimulatory molecule, has been proven to block T cell activation by competing with CD28 for binding to CD80/CD86. A study indicated that the recombinant fusion protein (CTLA4IgG) can significantly block the expression of CD80/CD86 on the surface of macrophages, thereby significantly reducing lymphocyte infiltration and improving the SGs function [74]. In addition, another study showed that transplanted effective-mononuclear cells can reduce the infiltration of CD4+ T lymphocytes in the SGs of NOD mice by inducing M2 macrophage polarization, thereby restoring the salivary secretion function of SGs [94]. Therefore, novel cell therapy holds extremely broad prospects for the treatment of SS. However, this treatment method still has significant limitations. For example, the large-scale production process is not yet mature, and the cost of a single treatment may be as high as several hundred thousand dollars.

Molecular Targeted Therapy

The protein molecules in macrophages are key driving factors in their involvement in SS pathogenesis. Therefore, how to directionally regulate the expression of protein molecules in macrophages has long been the focus of research on macrophage-targeted therapy for SS. Previous studies have clearly indicated that the promotion of SS disease progression by macrophages is associated with the dysregulated expression of CX3CR1 and Mer receptors. In recent years, numerous studies have demonstrated that regulating macrophage phenotypes based on these targets is of great significance for the treatment of SS [14,66].

Dietary Therapy

In fact, SS and diabetes share numerous similarities in their pathogenesis. For example, NOD mice, a non-obese diabetic model, exhibit infiltration of lymphocytes and macrophages in the LGs and SGs along with typical symptoms of dry mouth and dry eyes, making them frequently utilized as an animal model for SS research. Additionally, a study indicates that a high-fat diet can reduce tear secretion by promoting the infiltration of M1 macrophages into the lacrimal glands [95]. More notably, a study has demonstrated that a gluten-free diet therapy can simultaneously enhance salivary flow, reduce focus scores, and mitigate the infiltration of macrophages and T lymphocytes in NOD mice [96]. Therefore, developing healthy eating habits is as important as a pharmacological intervention for the improvement of SS-related symptoms.

## Conclusions

Macrophages are key immune cells that bridge innate and adaptive immunity, playing a critical role in the pathogenesis of SS. During SS-related inflammation, macrophages infiltrate the EGs prior to lymphocyte infiltration, and they are involved in the clearance of apoptotic cells, the maturation and migration of lymphocytes, and the fibrosis of exocrine glands, among other processes. Given their early infiltration, therapeutic modulation of macrophages may prevent subsequent lymphocyte-driven autoimmunity. Therefore, macrophage-targeted therapy is of great significance for alleviating the pathological progression of EGs in SS patients. Unfortunately, macrophage-based therapeutic strategies for SS have rarely been studied. M2 macrophages have been considered in previous studies to be beneficial for the resolution of inflammation and functional recovery of EGs in SS patients. Therefore, how to directionally induce macrophage differentiation safely, efficiently and with low side effects remains an urgent problem to be solved. Undoubtedly, additional mechanisms regulating macrophage behavior in SS likely remain to be elucidated. Therefore, the more detailed mechanisms of macrophages and how to target macrophages for the treatment of SS are both important research schemes in the future.
